# LED pumped polymer laser sensor for explosives

**DOI:** 10.1002/lpor.201300072

**Published:** 2013-10-08

**Authors:** Yue Wang, Paulina O Morawska, Alexander L Kanibolotsky, Peter J Skabara, Graham A Turnbull, Ifor D W Samuel

**Affiliations:** Organic Semiconductor Centre, SUPA, School of Physics and Astronomy, University of St AndrewsNorth Haugh, St Andrews, KY16 9SS, UK; WestCHEM, Department of Pure and Applied Chemistry, University of StrathclydeThomas Graham Building, Glasgow, G1 1XL, UK

**Keywords:** distributed feedback laser, organic semiconductor, indirect electrically pumping, triplet exciton, explosive sensing

## Abstract

A very compact explosive vapor sensor is demonstrated based on a distributed feedback polymer laser pumped by a commercial InGaN light-emitting diode. The laser shows a two-stage turn on of the laser emission, for pulsed drive currents above 15.7 A. The ‘double-threshold’ phenomenon is attributed to the slow rise of the ∼30 ns duration LED pump pulses. The laser emits a 533 nm pulsed output beam of ∼10 ns duration perpendicular to the polymer film. When exposed to nitroaromatic model explosive vapors at ∼8 ppb concentration, the laser shows a 46% change in the surface-emitted output under optimized LED excitation.

## 1. Introduction

Organic semiconductors are attractive laser materials due to their high gain, broad spectra and simple fabrication, enabling the development of low cost coherent light sources with a wide wavelength-tuning range 1–5. Recent applications that have been developed for this family of lasers include spectroscopy 6,7 and chemosensing 8–12. Organic semiconductor lasers (OSLs) are particularly promising for sensing explosive vapors, and have the potential for high sensitivity and rapid response 9,11,12. The principle behind this type of explosive sensor is that the presence of the strongly electron-deficient nitroaromatic explosive molecules quenches light emission in the organic gain medium, increasing the laser threshold and reducing the output light. Compared with polymer sensors based on fluorescence, the quenching of stimulated emission in the laser sensors can give rise to a significant increase in sensitivity and response speed. The highest sensitivity is achieved when the vapors switch the laser off to just below threshold; a good understanding of the polymer laser operation around threshold is therefore very important.

There are currently many different techniques used to detect explosives, mainly categorised by electromagnetic imaging, trained animals and spectrometry 13–16. The rise in international terrorism has required both sensitive and low-cost detecting methods. Mass and ion mobility spectrometry 16, gas chromatography 14, surface-enhanced Raman spectroscopy 13 along with fluorescent polymers 15 have been used for the detection and quantification of trace explosive chemicals. Substantial work has been done towards improving the detection limits for trinitrotoluene (TNT) and its relatives, which have reached femtogram levels, particularly by using mass spectrometry 17 and fluorescent conjugated polymers 18. One other criterion for a practical explosive vapor sensor is portability. To make conjugated polymer laser sensors meet this requirement, the main challenge is to reduce the size of the pump source. Direct electrical pumping of OSLs has so far not been achieved, and most OSLs require a second laser as the excitation source 5. A recent breakthrough was the demonstration of indirect electrical pumping of polymer lasers by InGaN light emitting diodes (LEDs) 19,20. In this work, we demonstrate the first explosive vapor sensor based on an LED-pumped polymer laser. We study the operation of the device around laser threshold in detail, and discover a two-stage turn on of the laser arising from the long pulse operation of the pump LED. The laser shows a maximum sensing efficiency of 68% in 90 seconds, demonstrating a compact approach to the sensitive detection of explosive vapors.

## 2. Laser design and operation

**Figure 1:**
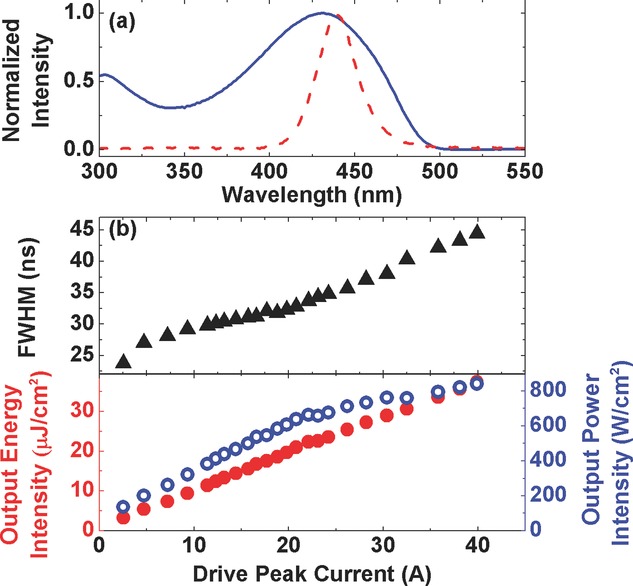
(a) Absorption (solid line) spectrum of a BBEHP-PPV thin film along with emission spectrum of a royal-blue LUXEON LED (dashed); (b) full width at half maximum (triangles) of optical pulse output from a LUXEON LED operating at 20 Hz, with corresponding energy intensity (closed circles) and power intensity (open circles) as a function of peak driving current.

For the polymer lasers we used a highly efficient light-emitting polymer, poly[2,5-bis(2′,5′-bis(2′′-ethylhexyloxy) phenyl)-*p*-phenylene vinylene] (BBEHP-PPV) with a weight-averaged molecular weight of 341,300 g/mol as the gain and sensing medium. This polymer has been previously used for detection of 2,4,6-trinitrotoluene (TNT) and 2,4-dinitrotoluene (DNT) and reported to have good gain properties and low laser threshold 9,20. The polymer was synthesized according to the procedure in ref. 20. Thin films were prepared by spin-coating a solution of 15 mg/ml of the polymer in chlorobenzene onto fused silica substrates. The film thicknesses were measured using a Veeco Dektak 150 surface profiler. The absorption spectrum of BBEHP-PPV is shown in Fig.1(a), along with the emission spectrum of the LED. The photoluminescence (PL) spectrum from the polymer film has two distinctive peaks in the green region at 495 nm and 530 nm, and the photoluminescence quantum yield (PLQY) was measured to be 84% using an integrating sphere 21 in a Hamamatsu Photonics C9920–02 measurement system with an excitation at 450 nm 22. As reported previously, the optical gain is peaked at 533 nm, close to the 0–1 vibronic transition in the PL spectrum.

To match the polymer film absorption (peaked at 431 nm), we used a royal-blue LED (Philips LUMILEDS LUXEON® Rebel LED), with a peak emission wavelength of 448 nm. The light emitting area of the LED is 1.3 mm by 1.3 mm, and under a continuous-wave (cw) operation the typical radiometric power from the LED (with its domed plastic cover in place) was specified to be 875 mW at a driving current of 700 mA, i.e. approximately 50 W/cm^2^. In order to pump the polymer laser, the InGaN LED was driven with nanosecond pulses generated by a compact laser diode driver (module PCO-7110–120–15, Directed Energy Inc.). The trigger signal for the driver was provided by a function generator, which also controlled the repetition frequency of the pulses. For pumping polymer lasers we remove the plastic cover on the LED. The output pulse energy from the LED was then measured with a calibrated energy meter for a range of driving currents at 20 Hz repetition rate, which is plotted in Fig.1 (b). As the peak drive current increases to 40 A, the energy density at the light-emitting surface of the LED reached as high as 37 μJ/cm^2^ with a pulse duration of 45 ± 2 ns (i.e. a peak power density of 840 W/cm^2^). The pulsed current damage threshold was found to be above 50 A (and there were some variations between LEDs). The maximum intensity generated by the pulsed LEDs was approximately 1000 W/cm^2^, 20-times higher than the output intensity under cw operation mode.

A one-dimensional fused silica grating was fabricated using two-beam laser interference in photoresist followed by a reactive-ion etching process. Surface-emitting DFB lasers were fabricated by spin-coating a thin film of BBEHP-PPV onto the fused silica grating. To achieve the lowest threshold for the polymer lasers, the feedback wavelength was optimized to be close to the ASE peak (533 nm) by varying the film thickness, and hence the effective refractive index, 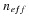, of the air-polymer-silica waveguide, according to the Bragg condition when the laser emits in the perpendicular direction to the grating surface, 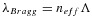, where Λ is the grating period. The grating used in this work was a second-order grating with a period of 355 ± 5 nm and an average groove depth of 55 nm ± 10 nm. In the initial laser threshold measurements, a Nd:YAG pumped optical parametric oscillator (OPO) at a wavelength of 450 nm (repetition rate of 20 Hz and pulse duration of 4 ns) was used as the optical excitation source. The surface emission from the polymer lasers was monitored with a fiber-coupled Chromex spectrograph equipped with a charge-coupled device (CCD) detector (Andor DV420-OE). With an excitation spot shaped into 0.97 mm in diameter, the optimized BBEHP-PPV laser threshold was measured to be as low as 118 W/cm^2^ with the laser wavelength at 533 nm, for a polymer film thickness of 170 nm. The polymer laser has an operational lifetime to half-power in air of ∼2 × 10^5^ pulses 23.

**Figure 2:**
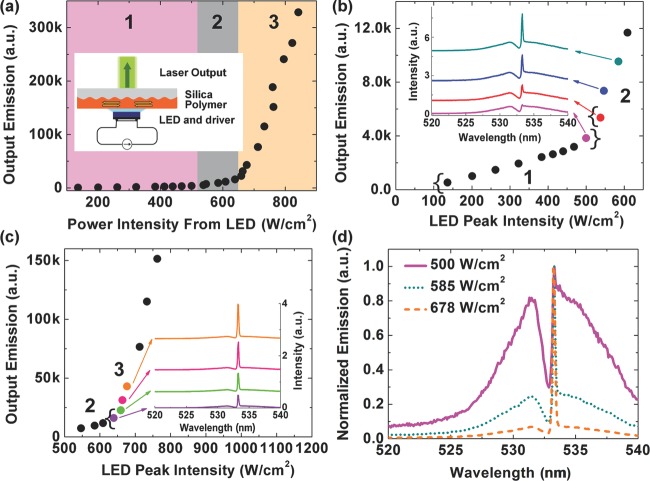
(a) Polymer output emission intensity at the wavelength of 533 nm as a function of LED power intensity. Inset: schematic drawing of an LED-pumped DFB polymer laser; (b) and (c) intensity of lasing peak as a function of LED power intensity. The graphs also show the regions of pump intensity of Fig.2(a). Region 1: below 500 W/cm^2^; region 2: 538 – 609 W/cm^2^; region 3: above 640 W/cm^2^. Inset: laser emission spectra at different pump power intensities; (d) overlap of normalized emission spectra at three different LED power intensities 500 W/cm^2^ (solid line), 585 W/cm^2^ (dotted line) and 678 W/cm^2^ (dashed line).

We then went on to investigate the second order DFB laser using the LUXEON LED as the pump source. With the plastic lens removed from the LED, the maximum power density at the light-emitting surface can be efficiently accessed by the polymer laser. As the LED power was increased we observed the laser emission turn on in two stages. The shaded regions in Fig.2(a) represent three regions of operation with respectively low, medium and high power density from the LED. At a low power density from the LED (below 500 W/cm^2^), a pronounced Bragg dip is observed at 532.8 nm in the light diffracted out the surface of the corrugated polymer film. The Bragg dip arises from a photonic stop-band for waveguided modes, which inhibits the emission and propagation of waveguided PL in the corrugated film 24. With the power density increased to 500 W/cm^2^ (corresponding to a peak driving current of 15.7 A), a sharp peak of linewidth 0.2 nm appears at a wavelength of 533.2 nm at one band-edge of the Bragg dip, as shown in Fig.2(b). The spectral change is accompanied by an abrupt change in the slope of the output intensity versus the input intensity, indicating the onset of band-edge DFB lasing. Interestingly when the LED power is further increased above 640 W/cm^2^, a second abrupt slope change in the output versus input curve is observed and the narrow lasing peak dominates the emission spectra, as shown in Fig.2(c). The spectra from these three pump regions are directly compared in Fig.2(d). It is clear that the laser linewidth from region 2 and 3, with and without the Bragg scattering shoulder, remains the same.

**Figure 3:**
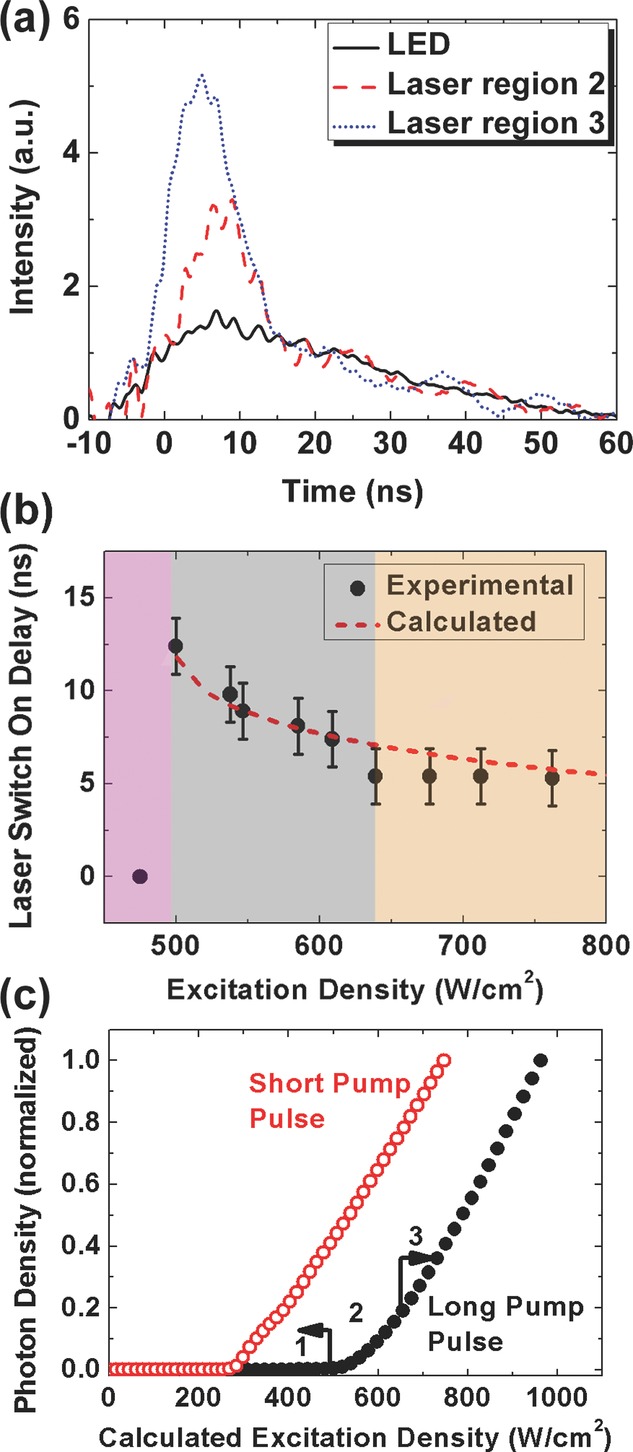
(a) Dynamics of output pulses from LED (solid line) and polymer laser with the pump intensity in region 2 (dashed line) and 3 (dotted line) respectively; (b) the delay between LED pulse and polymer laser in pump intensity region 2 and 3 (the delay is 0 in region 1 where the LED intensity is below the laser threshold). The dashed curve shows the fitted laser turn-on time using the rate-equation model; (c) laser threshold modeling with short and long excitation pulses in the polymer laser system.

To gain more insight into the operation in these three regions in Fig.2 we next measured the dynamics of the LED and polymer light emission using a fast photodiode (Standa HSP-V2), with a bandwidth of > 2 GHz and rise and fall time of < 150 ps. Figure3(a) shows example measurements of the laser dynamics when operating in region 2 and 3. Correlated with the changes in spectrum/output intensity, the dynamics also show three regions of operation (Fig.3(b)). In region 1, for pump intensities below threshold, the polymer emission dynamics closely matched those of the LED, indicating that the output emission consists only of fluorescence. In region 2, however, the polymer emission shows a spike in intensity on top of the fluorescence pulse, delayed ∼10s ns after the turn-on of the LED. This spike indicates a (delayed) onset of laser emission, which causes the first observed slope change. As the pump intensity increases through region 2 the build-up delay of stimulated emission decreases from 12 to 7 ns, see Fig.3(b). Finally in region 3, with even higher excitation intensity, the laser starts promptly (∼5 ns) after the initial rise of the LED pulse, and more effectively depletes the population inversion to give the second slope change. At a peak driving current of 24 A, the optical pulse width from the polymer laser was 11 ± 1 ns (FWHM), while the pump pulse from the LED was 36 ± 2 ns (FWHM). The laser terminated approximately 15 ns before the LED pulse reduced to its half maximum. We also note that this ‘double-threshold’ phenomenon was not observed when pumping with the short pulses (4 ns) of the OPO. This suggests that the increased threshold density and two-stage turn on of the LED-pumped polymer laser are due to the relatively long pump pulses and may relate to an accumulation of triplets in the laser.

## 3. Model of laser dynamics

Excited-state absorption and scattering are known to affect the performance of organic semiconductor lasers 25,26 and limit them to pulsed operation 27; to assess these possible effects on the LED-pumped laser output, we modelled the laser dynamics with the following rate equations 25,26

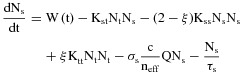


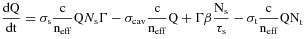


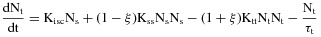


Here, *N*_s_ and *N*_t_ are the singlet and triplet exciton densities and *Q* is the photon density in the lasing mode. The LED excitation rate, *W*(t), is defined as 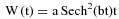. The sech-squared pulse is multiplied by a factor *t* to give an asymmetric pulse shape with a slower fall time than rise time, to approximate the shape of the experimental LED pulse; the value of *b* was set to give a FWHM of 35 ns or 2 ns. The triplet lifetime *τ*_t_ was set to a value ( = 25 μs 25) much longer than the measured singlet lifetime τ_s_ = 550 ps. The effective refractive index *n*_eff_ and transverse modal confinement factor *Γ* were both evaluated at the lasing wavelength, 533 nm, and the spontaneous emission factor, *β*, was assigned a value of 10^−4^ 25. The parameters *K*_ss_, *K*_st_ and *K*_tt_ are the singlet-singlet, singlet-triplet and triplet-triplet annihilation rates respectively, and *K*_isc_ is the intersystem crossing yield. Their values used in the model are presented in Table1, and are typical values for PPV derivatives 26,28. *ξ* = 0.25 is the assumed probability of singlet excitons produced per annihilation reaction, determined by spin statistics 26. The triplet absorption cross section *σ*_t_, stimulated emission cross section *σ*_s_ and total cavity loss *σ*_cav_ were used as fitting parameters, constrained to give laser thresholds similar to those measured experimentally and to fit the laser turn on times in Fig.3(b). The values which were found to give the best fit across the data are given in Table1.

**Table 1:** Values of annihilation rates and model fitting parameters

Stimulated emission cross section (σ_s_)	Triplet absorption cross section (σ_t_)	Cavity Loss (σ_cav_)	Singlet-singlet annihilation rate (K_ss_) 26	Singlet-triplet annihilation rate (K_st_) 26	Triplet-triplet annihilation rate (K_tt_) 26	Inter-system crossing rate (K_isc_) 26
1.8 × 10^−16^ cm^2^	4.3 × 10^−17^ cm^2^	2.35/cm	6 × 10^−9^ cm^3^/s	1.5 × 10^−9^ cm^3^/s	1 × 10^−14^ cm^3^/s	5 × 10^7^ /s

Figure3(c) shows the modeled power characteristics of the laser, pumped by both short pulses (FWHM = 2 ns) and also long pulses (FWHM = 35 ns). We find that the long pump pulses result in a higher laser threshold, and give a more gradual change in the slope of the laser emission just above threshold, both consistent with the experimental results. We attribute quantitative differences between the shape of the experimental and modeled power characteristics to the approximate function *W*(t) used in the calculations. The calculated turn-on time of the laser pulse is plotted as a dashed line in Fig.3(b). We find that the model can very successfully replicate the experimentally observed dynamics through pump region 2 and 3 of the laser operation. The values of fitting parameters used in the model are realistic for organic semiconductors; *σ*_s_ is close to the value measured for MEH-PPV 28, *σ*_cav_ is similar to the measured waveguide loss in BBEHP-PPV 20, while *σ*_t_, is comparable to the value used in 25. We note that the model parameters which most strongly affect the laser operation around threshold (for our LED pump pulses) are triplet absorption and singlet-triplet annihilation, indicating that these are responsible for the experimentally observed power characteristics of the laser.

In addition we measured the BBEHP-PPV laser output beam divergence by measuring the polymer laser beam profile at various distances for an LED power of 842 W/cm^2^. The laser beam divergence was found to be 0.1 mrad in the direction perpendicular to the plane of the fan-shaped output. The radiation pattern of the blue LED is Lambertian. Therefore, with the LED pumped polymer laser, we successfully convert an incoherent, divergent light source into a coherent and highly directional laser source.

## 4. Explosive vapor sensing

**Figure 4:**
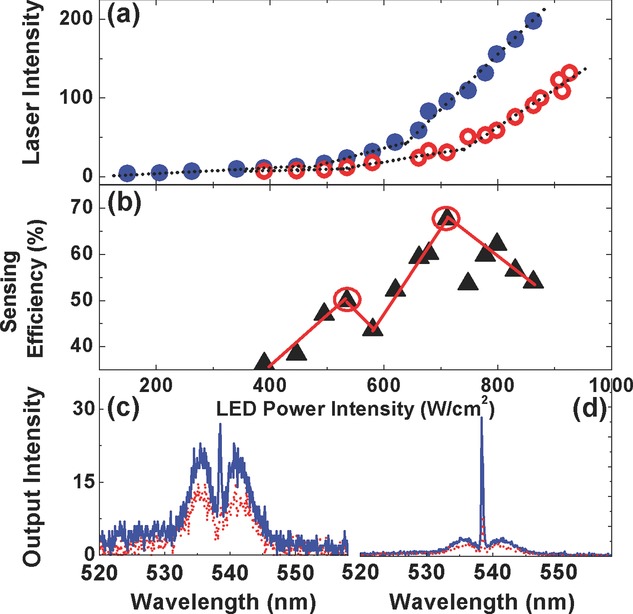
(a) Power characteristics of an InGaN LED pumped BBEHP-PPV laser before exposure (closed circles) and after a 90-second exposure (open circles) to the model explosive 1,4-DNB; (b) the sensing efficiency as a function of LED power intensity, the solid lines are guides for the eye. The two circles highlight two ‘thresholds’ of the DNB-exposed laser. The emission spectra of the unexposed (solid line) and exposed (dotted line) laser at these two threshold intensities are plotted respectively in (c) and (d).

Having shown that the LED pumped polymer laser can operate well above threshold, we then investigated the potential of using the hybrid laser as a sensor to detect vapor of a model nitro-aromatic explosive, 1,4-dinitrobenzene (DNB). As a benchmark for laser sensing of explosives, PL sensing measurements using BBEHP-PPV thin films were first performed. The BBEHP-PPV films were placed inside an optical chamber connected to two gas ports allowing flow of either DNB-containing or clean nitrogen 12. The films were excited at 450 nm and the emission spectra were monitored by the fibre-coupled CCD. Sensing efficiency is defined as the change in the intensity normalized to the original intensity. After 90 s continuous exposure to the DNB vapor, the PL intensity of a 150 nm thick film decreased by 6% (i.e. a sensing efficiency of 6%). Full recovery of the PL emission from the exposed film was observed with a 3-minute clean-nitrogen purge. We then investigated the use of an InGaN LED pumped BBEHP-PPV laser as a DNB vapor sensor. The laser thresholds were measured before and after a 90-second exposure to the DNB vapor, while the laser emission spectra were recorded simultaneously. Figure4(a) shows the laser output as a function of LED power density before and after DNB exposure. After 90 seconds, the first lasing threshold was seen to increase from 13 A (446 W/cm^2^) to 17 A (535 W/cm^2^), an increase of 20%. The second threshold increased from 20 A (620 W/cm^2^) to 26 A (711 W/cm^2^). A lower output-input slope of the exposed laser was observed, attributed to an extra loss in the gain medium induced by the quenchers. The sensing efficiency of the laser increased through region 2 from below 40% to a maximum sensing efficiency of 68% at the second threshold of the DNB-exposed laser, as shown in Fig.4(b). The laser based sensor therefore responded much more strongly than the sensors based on spontaneous emission, and this could be used to detect explosives more quickly. In addition, we observed the laser threshold and output power to recover to their original values within 3 minutes of exposure of clean nitrogen gas.

**Figure 5:**
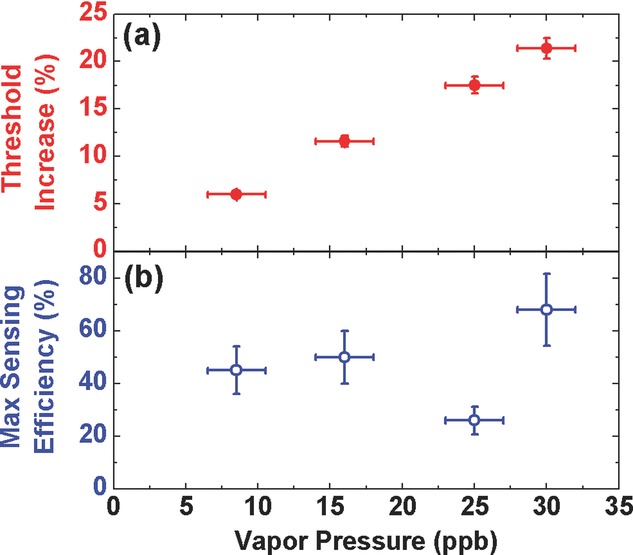
(a) Measured increase in threshold of the polymer laser following exposure to different DNB vapor pressures; (b) maximum sensing efficiency of the laser sensor (for the optimum pump intensity) as a function of DNB vapor pressure.

To investigate the sensitivity of the LED pumped laser sensor, we measured both the laser threshold and attenuation of the emission following 90 s exposure to a range of DNB vapor concentrations. The DNB concentration was varied over approximately a factor of 4 (giving an estimated range of 8–30 ppb) by diluting a nitrogen flow carrying DNB vapor with a clean nitrogen source. We can see from Fig.5(a) that the change in the polymer laser threshold following DNB exposure decreases when the laser is exposed to lower vapor pressure. When the laser was exposed to ∼8 ppb DNB vapor for 90 seconds, we observed a 6% increase in laser threshold and a maximum sensing efficiency of 46%, see Fig.5(b). Under the same exposure conditions with a BBEHP-PPV film of similar-thickness, we observed no change in the PL. Once again, the laser sensor has a much higher sensitivity compared to sensors based on PL.

## 5. Conclusions

In conclusion, we have demonstrated an InGaN LED pumped BBEHP-PPV laser sensor. The polymer laser was pumped by a commercial blue LED with a peak wavelength appearing at 533 nm for pulsed drive currents above 15.7 A. A two-stage turn-on of the laser was observed, and explained using a model including the effects of triplets. The polymer laser sensor can detect 8 ppb of the model explosive vapor after only a 90-second exposure of the polymer laser to the explosive vapor. The lasing threshold increases causing the laser emission intensity to drop dramatically. These highly sensitive and inexpensive LED-pumped polymer laser sensors could be used in humanitarian demining, complementing existing technologies such as ground-penetrating radar leading to an improvement in the detection of hazardous objects.
